# [Retracted] Mechanism of IL‑8‑induced acute lung injury through pulmonary surfactant proteins A and B

**DOI:** 10.3892/etm.2025.12875

**Published:** 2025-04-28

**Authors:** Yinong Yang, Qing Li, Feng Tan, Jun Zhang, Wu Zhu

Exp Ther Med 19:287–293, 2020; DOI: 10.3892/etm.2019.8192

Following the publication of the above article, a concerned reader drew to the Editor's attention that the backgrounds surrounding the surfactant protein (SP)-A and -B protein bands shown in Fig. 6B and C were surprisingly similar comparing between the gel slices; moreover, potential anomalies associated with the bands themselves were also identified.

After having conducted an internal investigation of the data in this paper, the Editor of *Experimental and Therapeutic Medicine* agrees that the presentation of the bands in Fig. 6B and C was potentially anomalous; therefore, the Editor has decided that this article should be retracted from the publication on the grounds of a lack of confidence in the data. The authors were asked for an explanation to account for these concerns, but the Editorial Office did not receive a reply. The Editor sincerely apologizes to the readership for any inconvenience caused, and we thank the reader for bringing this matter to our attention.

